# Correction: An engineered third electrostatic constriction of aerolysin to manipulate heterogeneously charged peptide transport

**DOI:** 10.1039/d2sc90101c

**Published:** 2022-05-20

**Authors:** Hongyan Niu, Meng-Yin Li, Yi-Lun Ying, Yi-Tao Long

**Affiliations:** State Key Laboratory of Analytical Chemistry for Life Science, School of Chemistry and Chemical Engineering, Nanjing University Nanjing 210023 P. R. China; Chemistry and Biomedicine Innovation Center, Nanjing University Nanjing 210023 P. R. China yilunying@nju.edu.cn

## Abstract

Correction for ‘An engineered third electrostatic constriction of aerolysin to manipulate heterogeneously charged peptide transport’ by Hongyan Niu *et al.*, *Chem. Sci.*, 2022, **13**, 2456–2461, https://doi.org/10.1039/D1SC06459B.

The authors regret an error in the spelling of Meng-Yin Li’s name in the original manuscript. The correct spelling is as shown in this Correction article.

The Royal Society of Chemistry apologises for these errors and any consequent inconvenience to authors and readers.

## Supplementary Material

